# The oribatid mite genus *Benoibates* (Acari, Oribatida, Oripodidae)

**DOI:** 10.3897/zookeys.442.8361

**Published:** 2014-09-23

**Authors:** Sergey G. Ermilov, Olman Alvarado-Rodríguez, Jenő Kontschán, Axel P. Retana-Salazar

**Affiliations:** 1 Tyumen State University, Tyumen, Russia; 2 Centro de Investigación en Estructuras, Microscópicas (CIEMIC), Ciudad de la Investigación, Universidad de Costa Rica, San José, Costa Rica; 3 Center for Agricultural Research, Plant Protection Institute, Hungarian Academy of Sciences, Budapest, Hungary

**Keywords:** Oribatid mites, *Benoibates*, redescription, key, Costa Rica

## Abstract

Two species of oribatid mites of the genus *Benoibates* (Oribatida, Oripodidae), i.e., *Benoibates bolivianus* Balogh & Mahunka, 1969(a) and *Benoibates minimus* Mahunka, 1985, are recorded for the first time in Costa Rica. Both are redescribed in details, using drawings, images and SEM micrographs, on the basis of Costa Rican specimens. An identification key to the known species of *Benoibates* is given.

## Introduction

*Benoibates* (Acari, Oribatida, Oripodidae) is a genus of oribatid mites that was proposed by Balogh (1958) with *Benoibates flagellifer* Balogh, 1958 as type species. Currently, it includes 12 species, distributed in the Neotropical region (seven species), U.S.A. (two species), Ethiopian region (two species) and Polynesia (one species) (data summarized by Subías 2004, updated 2014).

Subías (2004, updated 2014) includes additionally three genera as junior synonyms in *Benoibates*: *Exoripoda* Woolley 1961 (with two species: *Exoripoda excavatus* Woolley, 1961; *Exoripoda suramericanus* Mahunka, 1983), *Haploripoda* Balogh & Mahunka, 1967 (with one species: *Haploripoda reductus* Balogh & Mahunka, 1967) and *Reductoripoda* Mahunka & Palacios-Vargas, 1996 (with one species: *Reductoripoda absoluta* Mahunka & Palacios-Vargas, 1996). *Exoripoda* was discribed by Woolley (1961) on bases of the presence of one pair of adanal setae (versus two pairs in *Benoibates*); *Haploripoda* – Balogh and Mahunka (1967) on bases of the presence of one pair of genital setae (versus two pairs in *Benoibates*); *Reductoripoda* – Mahunka and Palacios-Vargas (1996) on bases of the presence of one pair of adanal setae and the absence of anal setae (versus two and one pairs present in *Benoibates*, accordingly). Subías probably is right, but we cannot support his opinion at this moment, because many oripodid genera were proposed on the basis of distinctions in the number of anogenital setae – see also different views on classification of genera (for example, Woolley 1961, 1966; Aoki and Ohkubo 1974; Balogh and Balogh 1992, 1999; Subías 2004). Hence, the full and detail revision of all taxa in the family Oripodidae is necessary in the future.

The main generic characters of *Benoibates* in Oripodidae are (summarized by Balogh 1958, Aoki and Ohkubo 1974; Balogh and Balogh 1990, including our additions): rostrum rounded; bothridial openings not covered by notogaster; body surface foveolate; anterior notogastral margin convex medially, transverse straight; 10 (exception 11) pairs on notogastral setae present; two pairs of genital, one pair of aggenital, two pairs of adanal and one pair of anal setae present, genital setae inserted in anterior part of genital figs, anal setae inserted in posterior part of anal figs; legs tridactylous.

In the course of proceeding taxonomic identification of oribatid mites from Costa Rica (Ermilov et al. 2014a, b), we have found two species of *Benoibates*, *Benoibates bolivianus* Balogh & Mahunka, 1969(a) (described from Bolivia) and *Benoibates minimus* Mahunka, 1985 (described from Antilles). Both species are recorded for the first time in Costa Rican fauna.

The original descriptions of *Benoibates bolivianus* and *Benoibates minimus* were based only on holotypes, and, hence, it is incomplete and brief (lacking information about the measures of morphological structures, leg setation and solenidia, morphology of gnathosoma; only dorsal and ventral sides of body are illustrated). We also notice that *Benoibates*-species are very similar morphologically, and species descriptions of this genus were brief. Therefore their supplementary descriptions are especially important now. The main goal of our paper is to present detailed redescriptions and illustrations of *Benoibates bolivianus* and *Benoibates minimus*, using drawings, images and SEM (Scanning Electron Microscopy) micrographs, of Costa Rican specimens.

The second goal of our paper is to present an identification key to the based on *Benoibates* known species.

## Materials and methods

### Material

***Benoibates bolivianus* Balogh & Mahunka, 1969**

Three specimens (male and two females), Costa Rica, 9°50'24"N, 83°53'17"W, Cartago, Dulce Nombre, Paraíso, Jardín Botánico Lankester, 1400 m a.s.l., in leaf litter in secondary forest, 14.V.2013, collected by O. Alvarado-Rodríguez and A.P. Retana-Salazar. Holotype (0-555-68, Hungarian National History Museum, Budapest) (see Balogh and Mahunka 1969a): Bolivia, “Guayaramerin, Beni, forest along the river Mamore, litter and wooden debris from the shady base of a low tree”, 26.XI.1966 (collected by J. Balogh, S. Mahunka and A. Zicsi).

***Benoibates minimus* Mahunka, 1985**

Five specimens (four males and one female), Costa Rica, 9°50'24"N, 83°53'17"W, Cartago, Dulce Nombre, Paraíso, Jardín Botánico Lankester, 1400 m a.s.l., in leaf litter in secondary forest, 14.V.2013, collected by O. Alvarado-Rodríguez and A.P. Retana-Salazar. Holotype (971-HO-84, Hungarian National History Museum, Budapest) (see Mahunka 1985): Antilles, “Anse La Raye, Pilori Pt., singling from under bark of coastal trees and sifting rotten material accumulated at tree bases and picking out animals”, 14.VII.1980 (collected by S. Mahunka).

### Methods

The specimens were mounted in lactic acid on temporary cavity slides for measurement and illustration. The body length was measured in lateral view, from the tip of the rostrum to the posterior edge of the ventral fig. The notogastral width refers to the maximum width in dorsal aspect. Lengths of body setae were measured in lateral aspect. All body measurements are presented in micrometers. Formulae for leg setation are given in parentheses according to the sequence trochanter–femur–genu–tibia–tarsus (famulus included). Formulae for leg solenidia are given in square brackets according to the sequence genu–tibia–tarsus.

General terminology used in this paper follows that of Grandjean (summarized by Norton and Behan-Pelletier 2009).

Drawings were obtained by a drawing tube using the Carl Zeiss transmission light microscope “Axioskop-2 Plus”. Images were obtained by an AxioCam ICc3 camera using the Carl Zeiss transmission light microscope “Axio Lab.A1”. SEM micrographs were obtained by the Jeol scanning electron microscope “JSM-6510 LV”.

## Results

### Redescription of the studied species

#### Benoibates bolivianus

Taxon classificationAnimaliaOribatidaOripodidae

Balogh & Mahunka, 1969(a)

**Figures 1–2.**
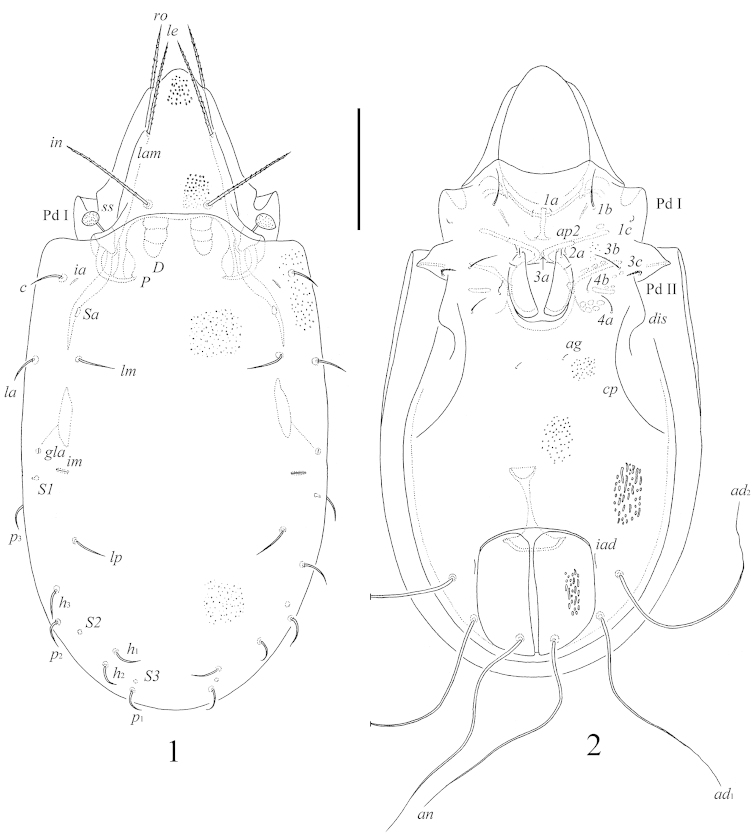
*Benoibates bolivianus* Balogh & Mahunka, 1969, Costa Rican specimen: **1** dorsal view **2** ventral view (gnathosoma and legs not illustrated). Scale bar 100 μm.

Figs 1 [Fig F2] [Fig F3] [Fig F4] [Fig F5] [Fig F6] –42

##### Diagnosis.

Body size: 514–597 × 265–332. Body surface weakly foveolate. Rostral, lamellar and interlamellar setae setiform, barbed; latter are longest. Bothridial setae short, clavate. Ten pairs of notogastral setae of medium size (24–32). Sacculi *Sa* large than other. Subcapitular setae *h* longer than *a* and *m*. Apodemes 2 connected medially and to anterior margin of genital aperture. Pedotecta II with one pointed tip anteriorly Genital and aggenital setae short. Anal and adanal setae very long, flagellate.

##### Description.

*Measurements*. Body length: 514–597 (three specimens); notogaster width: 265–332 (three specimens).

*Integument*. Body color yellowish brown to brown. Body surface weakly foveolate: prodorsum with distinct, round foveoles, larger in antero-medial part (up to 4) than in basal part (up to 1); notogaster, epimeral region, subcapitular mentum and gena, and genital figs with weak, round foveoles (up to 2); anogenital region and legs with distinct (except weak between genital and anal apertures), round or oval foveoles (up to 4), simultaneously also with longitudinal foveoles (length up to 16). Body surface of notogaster and ventral side covered by microgranular cerotegument (less than 1; visible only high magnification, ×1000).

*Prodorsum*. Rostrum weakly protruding, rounded. Lamellae (*lam*) located dorso-laterally, half length of prodorsum (measured in lateral view), without cusps. Translamella absent. Prolamellar lines (*plam*) thin, reaching the insertions of rostral setae and bend ventrally to meet the rostral margins. Sublamellar lines (*slam*) distinct, long. Sublamellar porose areas (*Al*) small, rounded (4–6). Keel-shaped ridges (*kf*) well developed. Rostral (*ro*, 55–61), lamellar (*le*, 77–86) and interlamellar (*in*, 94–106) setae setiform, barbed. Interlamellar setae long, reaching the insertions of lamellar setae. Bothridial setae (*ss*, 32–41) with short stalk (16–21) and clavate, barbed head (16–20). Exobothridial setae (*ex*, 16) thin, smooth.

*Notogaster*. Anterior notogastral margin weakly convex, trapezoid. Dorsophragmata (*D*) elongated, not reaching pleurophragmata (*P*). Notogastral shoulders rectangular in dorsal view, anterior margin almost transverse straight. Ten pairs of notogastral setae of medium size (24–32), setiform, indistinctly barbed (visible under high magnification, ×1000). Insertions of setae *h*_1_–*h*_3_ varies. Four pairs of sacculi developed: *Sa* largest, located postero-medially to setae *c*; *S1* – postero-laterally to lyrifissures *im*; *S2* – between setae *h*_2_ and *h*_3_; *S3* – anteriorly to *p*_1_. Lyrifissures *ia* located medially to setae *c*; *im* – between setae *lm* and *lp*, in transverse position; *ip* – laterally to *p*_1_; *ih* – anteriorly to *p*_1_; *ips* – between *p*_2_ and *p*_3_. Opisthonotal gland openings (*gla*) located antero-laterally to lyrifissures *im*.

*Gnathosoma*. Subcapitulum longer than wide (118–127 × 86–98). Subcapitular setae setiform, slightly barbed; *h* (53–61) longer than *a* and *m* (both 28–32). Setae *m* thinnest. Two pairs of adoral setae (*or*_1_, *or*_2_, 20) setiform, densely ciliate. Palps (length 77) with setation 0–2–1–3–9(+ω). Solenidion attached to eupathidium. Chelicerae (length 127–139) with one barbed setae (*cha*, 36–41), *chb* and their alveoli absent. Trägårdh’s organ (Tg) long, tapered.

*Epimeral and lateral podosomal regions*. Apodemes 1, 2, 3, sejugal and sternal apodemes distinct. Apodemes 2 (*ap2*) connected medially and to anterior margin of genital aperture. Sternal apodeme of medium size, not reaching the apodemes 2. Epimeral setal formula: 3–1–3–2. Centroventral setae *1a*, *2a*, *3a* smooth, other slightly barbed; *1b* (41–53) longer than *3b* (24–28), *4a* (20), *4b*, *3c* (16–18), *1c*, *2a* (10) and *1a*, *3a* (6–8). Setae *3c* thickest. Pedotecta I (Pd I) large, concave (measured in dorsal view) and scale-like (measured in lateral view); pedotecta II (Pd II) smaller, trapezoid, with one pointed tip anteriorly (measured in ventral view) and scale-like (measured in lateral view). Discidia (*dis*) elongated, weakly triangular. Circumpedal carinae (*cp*) distinct.

*Anogenital region*. Two pairs of genital (*g*_1_, *g*_2_, 10) and one pair of aggenital (*ag*, 8) setae setiform, thin, smooth. One pair of anal (*an*) and two pairs of adanal (*ad*_1_, *ad*_2_) setae (all 176–196) very long, flagellate. Lyrifissures *iad* located close to and parallel anal figs.

*Legs*. Median claw weakly thicker than two lateral claws; all with several minute barbs on dorsal side. Lateral claws with ventral tooth. Formulae of leg setation and solenidia: I (1–5–2–4–16) [1–2–2], II (1–5–2–4–13) [1–1–2], III (2–3–1–3–13) [1–1–0], IV (1–2–2–3–11) [0–1–0]; homology of setae and solenidia indicated in Table 1. Famulus short, straight, slightly dilated distally, truncated. Solenidia ω_2_ on tarsi I, ω_1_ and ω_2_ on tarsi II, σ on genua II, III of medium size, thickened, blunt-ended. Other solenidia long, setiform.

**Table 1.** Leg setation and solenidia of *Benoibates bolivianus* Balogh & Mahunka, 1969 (same data for *Benoibates minimus* Mahunka, 1985).

Leg	Trochanter	Femur	Genu	Tibia	Tarsus
I	*v*’	*d*, *(l)*, *bv*’’, *v*’’	*(l)*, σ	*(l)*, *(v)*, φ_1_, φ_2_	*(ft)*, *(tc)*, *(it)*, *(p)*, *(u)*, *(a)*, *s*, *(pv)*, *e*, ω_1_, ω_2_
II	*v*’	*d*, *(l)*, *bv*’’, *v*’’	*(l)*, σ	*(l)*, *(v)*, φ	*(ft)*, *(tc)*, *(it)*, *(p)*, *(u)*, *(a)*, *s*, ω_1_, ω_2_
III	*l*’, *v*’	*d*, *l*’, *ev*’	*l*’, σ	*l*’, *(v)*, φ	*(ft)*, *(tc)*, *(it)*, *(p)*, *(u)*, *(a)*, *s*
IV	*v*’	*d*, *ev*’	*d*, *l*’	*l*’, *(v)*, φ	*ft*’’, *(tc)*, *(p)*, *(u)*, *(a)*, *s*, *pv*’’

Roman letters refer to normal setae (*ε* to famulus), Greek letters to solenidia. Single prime (’) marks setae on anterior and double prime (’’) setae on posterior side of the given leg segment. Parentheses refer to a pair of setae.

**Figures 3–6. F2:**
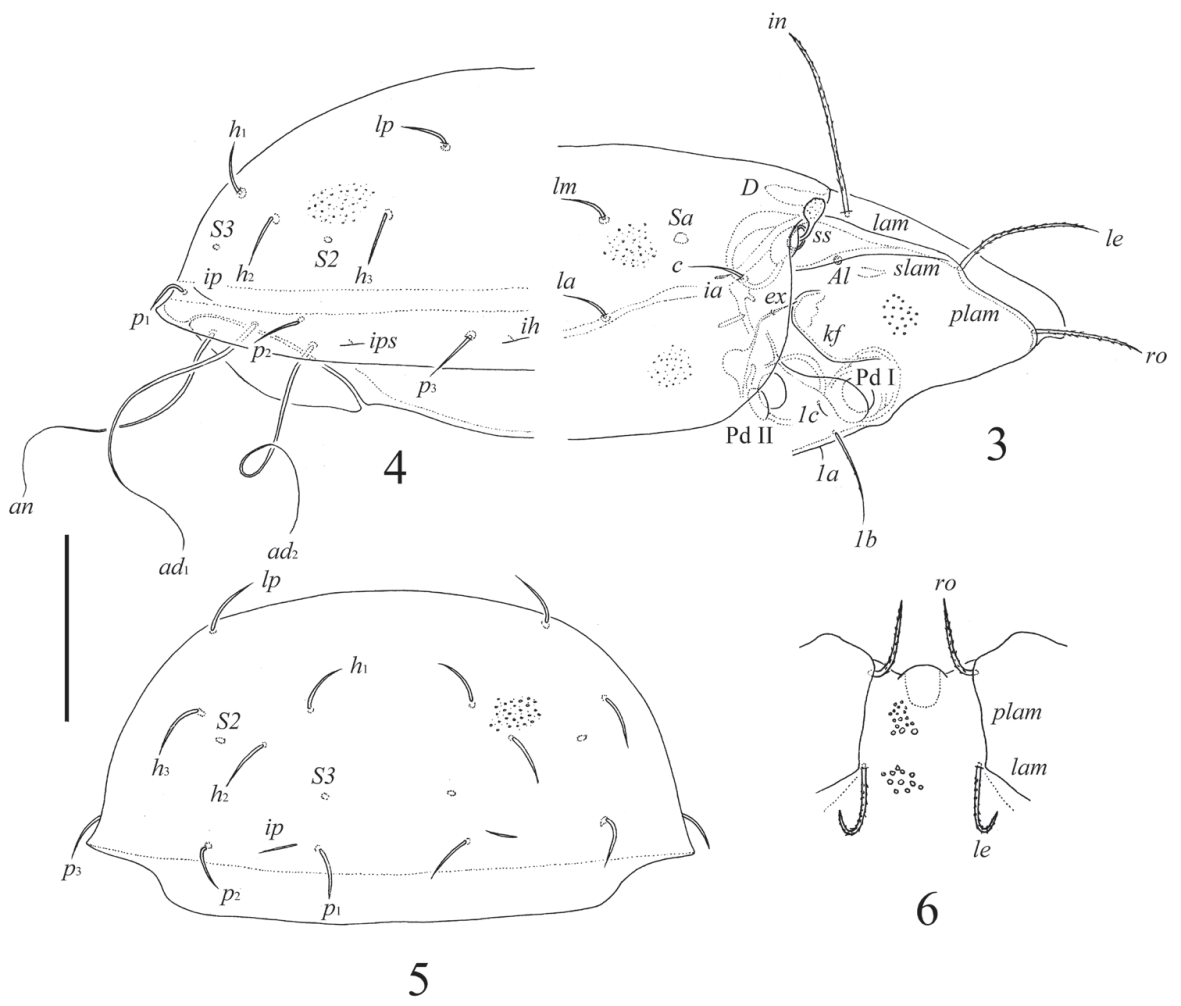
*Benoibates bolivianus* Balogh & Mahunka, 1969, Costa Rican specimen: **3** lateral view of prodorsum and anterior part of notogaster and pteromorph (gnathosoma and legs I, II not illustrated) **4** lateral view of notogaster **5** posterior view of notogaster **6** frontal view of prodorsum. Scale bar 100 μm.

**Figures 7–20. F3:**
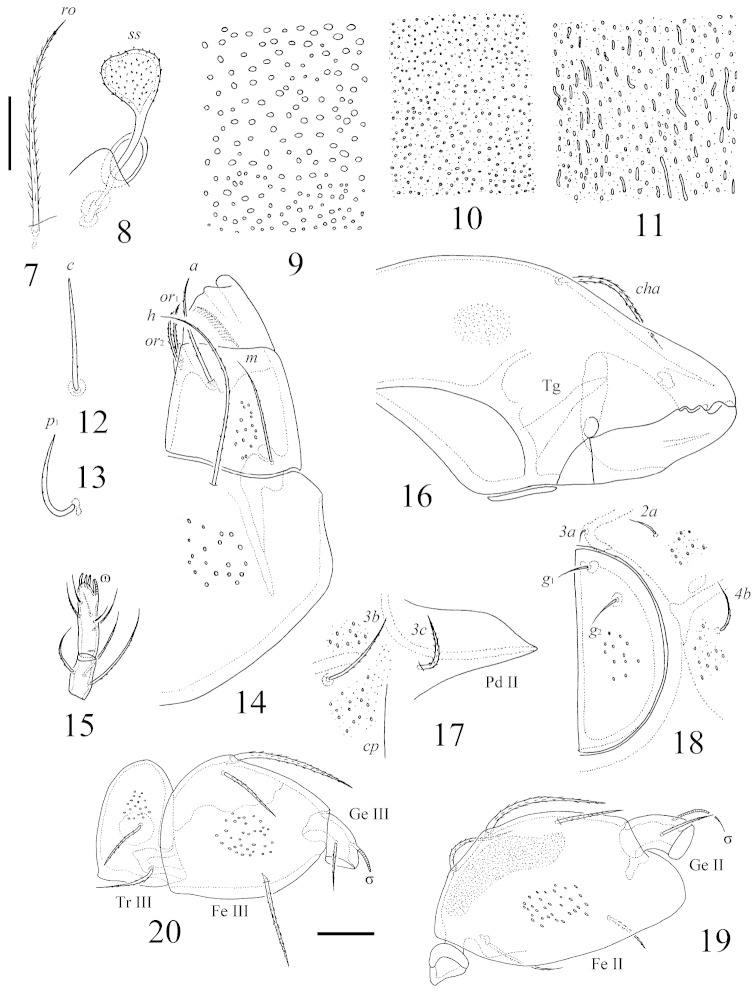
*Benoibates bolivianus* Balogh & Mahunka, 1969, dissected Costa Rican specimen: **7** rostral seta **8** posterior bothridial seta **9** foveoles in medio-basal part of prodorsum **10** foveoles in central part of notogaster **11** foveoles in lateral part of anogenital region **12** notogastral seta *c* **13** notogastral seta *p*_1_ **14** left part of subcapitulum, ventral view **15** tarsus and tibia of palp **16** antero-medial part of chelicera **17** pedotectum II, anterior part of circumpedal carina and epimeral setae *3b*, *3c* **18** left genital fig and epimeral setae *2a*, *3a*, *4b* **19** femur and genu of left leg II, paraxial view **20** trochanter, femur and genu of left leg III, antiaxial view. Scale bar 20 μm.

**Figures 21–31. F4:**
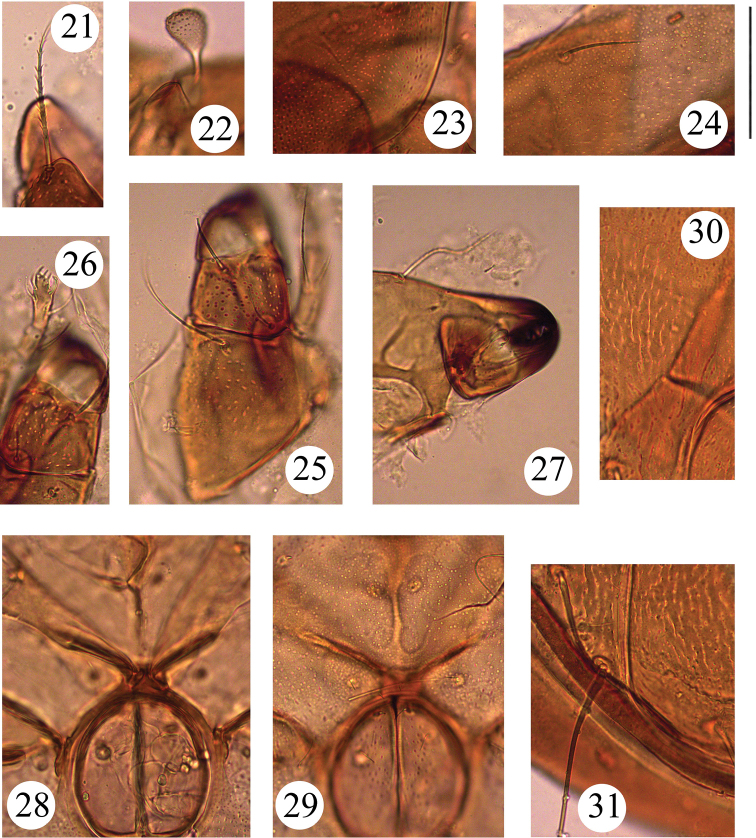
*Benoibates bolivianus* Balogh & Mahunka, 1969, dissected Costa Rican specimen, microscope images: **21** rostral seta **22** bothridial seta **23** foveoles in anterior part of pteromorph **24** notogastral seta *h*_3_ **25** left part of subcapitulum, ventral view, and medio-basal part of left palp **26** right rutellum and gena of subcapitulum, ventral view, and anterior part of right palp **27** antero-medial part of chelicera **28** genital figs and central part of epimeral region **29** genital figs and central part of epimeral region **30** antero-lateral part of right anal fig, insertion of adanal setae *ad*_2_, and foveoles in anogenital region **31** posterior part of right anal fig and insertions of anal and adanal setae. Scale bar 50 μm.

**Figures 32–36. F5:**
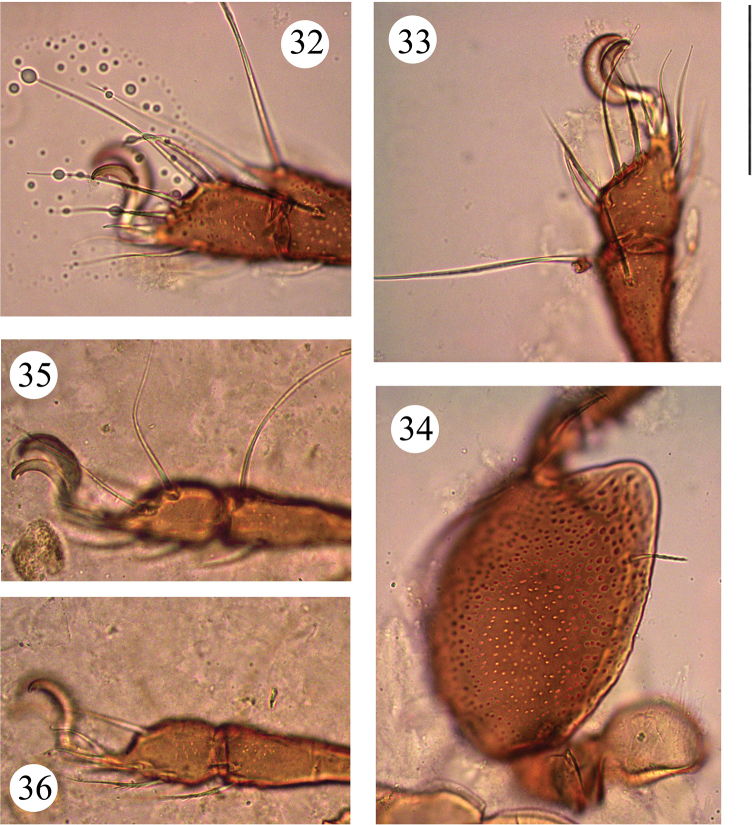
*Benoibates bolivianus* Balogh & Mahunka, 1969, dissected Costa Rican specimen, microscope images: **32** tarsus and anterior part of tibia of leg I, left, antiaxial view **33** tarsus and antero-medial part of tibia of leg II, right, antiaxial view **34** basal part of tibia, genu, femur and trochanter of leg II, right, antiaxial view **35** tarsus and antero-medial part of tibia of leg III, right, antiaxial view **36** tarsus and antero-medial part of tibia of leg IV, right, antiaxial view. Scale bar 50 μm.

**Figures 37–40. F6:**
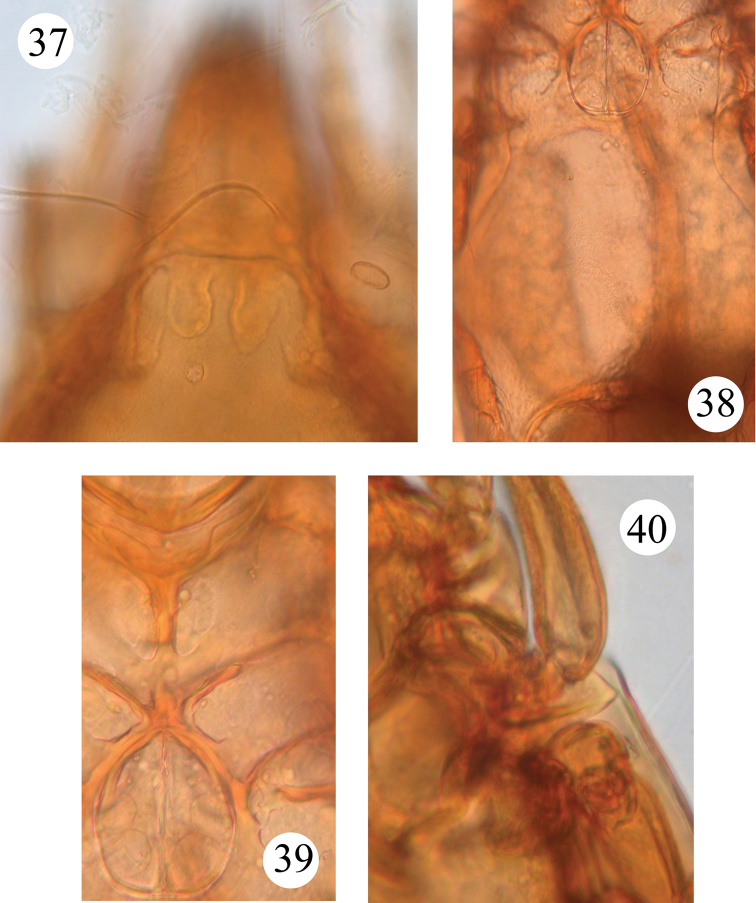
*Benoibates bolivianus* Balogh & Mahunka, 1969, holotype, microscope images: **37** dorsal view of prodorsum and anterior part of notogaster **38** ventral view of anogenital region **39** genital figs and central and left parts of epimeral region **40** ventral view of left pedotectum II.

**Figures 41–42.**
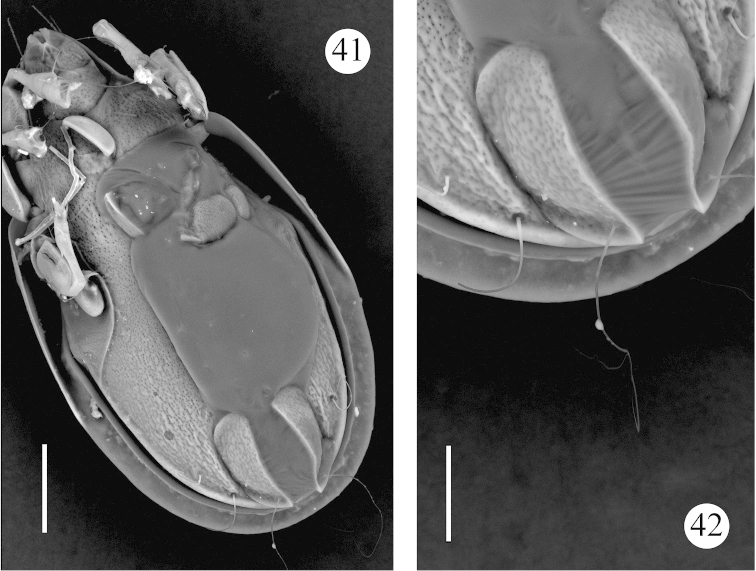
*Benoibates bolivianus* Balogh & Mahunka, 1969, Costa Rican specimen, SEM micrographs: **41** ventral view **42** ventral view of ano-adanal region. Scale bar 100 μm (**41**), 50 μm (**42**).

##### Remarks.

Costa Rican specimens of *Benoibates bolivianus* are similar in all morphological characters to Bolivian specimens from the original description (Balogh and Mahunka 1969a), except slightly shorter epimeral setae *1b*.

##### Distribution.

Neotropical region.

#### Benoibates minimus

Taxon classificationAnimaliaOribatidaOripodidae

Mahunka, 1985

**Figures 43–44.**
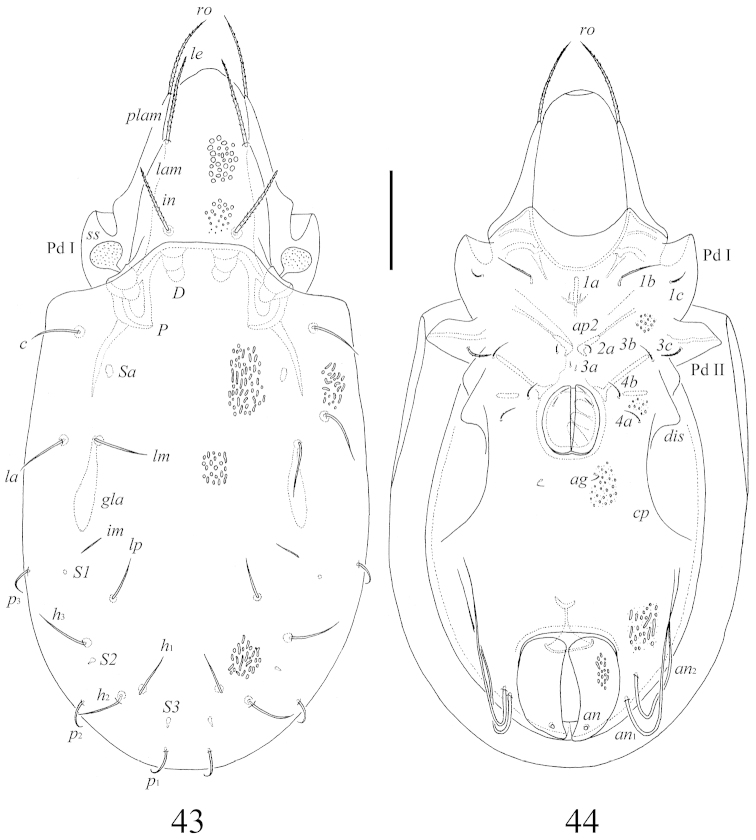
*Benoibates minimus* Mahunka, 1985, Costa Rican specimen: **43** dorsal view **44** ventral view (gnathosoma and legs not illustrated). Scale bar 50 μm.

Figs 43 [Fig F9] [Fig F10] [Fig F11] [Fig F12] [Fig F13] [Fig F14] –83

##### Diagnosis.

Body size: 344–481 × 176–249. Body surface heavily foveolate. Rostral, lamellar and interlamellar setae setiform, barbed; latter are longest. Bothridial setae short, clavate. Ten pairs of notogastral setae of medium size. Sacculi *Sa* large than other. Subcapitular setae *h* longer than *a* and *m*. Apodemes 2 connected medially and removed from the anterior margin of genital aperture. Pedotecta II with one pointed tip anteriorly Genital and aggenital setae short. Anal and adanal setae very long, flagellate.

##### Description.

*Measurements*. Body length: 344–481 (five specimens); notogaster width: 176–249 (five specimens).

*Integument*. Body color yellowish brown. Body surface heavily foveolate: prodorsum with distinct, round foveoles, larger in antero-medial part (up to 4) than in basal part (up to 1); epimeral region, subcapitular mentum and gena, and genital figs with round foveoles (up to 4); notogaster and anogenital region and legs with distinct (except weak between genital and anal apertures), round or oval foveoles (up to 4), simultaneously also with longitudinal foveoles (length up to 12). Body surface of ventral side covered by microgranular cerotegument (less than 1; visible only high magnification, ×1000).

*Prodorsum*. Rostrum weakly protruding, rounded. Lamellae located dorso-laterally, half length of prodorsum (measured in lateral view), without cusps. Translamella absent. Prolamellar lines thin, reaching the insertions of rostral setae and bend ventrally to meet the rostral margins. Sublamellar lines distinct, long. Sublamellar porose areas small, rounded (4). Keel-shaped ridges well developed. Rostral (36–49), lamellar (41–53) and interlamellar (49–61) setae setiform, barbed. Lamellar and interlamellar straight, blunt-ended. Bothridial setae (24–32) with short stalk (8–12) and larger, clavate, barbed head (16–20). Exobothridial setae (6–8) thin, smooth.

*Notogaster*. Anterior notogastral margin convex, trapezoid. Dorsophragmata elongated, not reaching pleurophragmata. Notogastral shoulders rectangular in dorsal view, anterior margin almost transverse straight. Ten pairs of notogastral setae of medium size (24–36; *p*_1_–*p*_3_ shorter, 20–24), setiform, smooth. Four pairs of sacculi developed: *Sa* largest, located postero-medially to setae *c*; *S1* – postero-laterally to lyrifissures *im*; *S2* – between setae *h*_2_ and *h*_3_; *S3* – anteriorly to *p*_1_. Lyrifissures *ia* not visible; *im* located between setae *lm* and *lp*, in diagonal position; *ip* – laterally to *p*_1_; *ih* – anteriorly to *p*_1_; *ips* – between *p*_2_ and *p*_3_. Opisthonotal glands located between setae *lm* and *lp*, but their openings not visible.

*Gnathosoma*. Subcapitulum longer than wide (86–98 × 61–69). Subcapitular setae setiform, slightly barbed; *h* (28–32) slightly thicker and longer than *a* and *m* (both 18–20). Two pairs of adoral setae (12) setiform, densely barbed. Palps (length 45–53) with setation 0–2–1–3–9(+ω). Solenidion attached to eupathidium. Chelicerae (length 90–102) with one barbed setae (*cha*, 28–36), *chb* and their alveoli absent. Trägårdh’s organ long, tapered.

*Epimeral and lateral podosomal regions*. Apodemes 1, 2, 3, sejugal and sternal apodemes distinct. Apodemes 2 connected medially and removed from the anterior margin of genital aperture. Sternal apodeme of medium size, not reaching the apodemes 2. Epimeral setal formula: 3–1–3–2. Centroventral setae *1a*, *3a* smooth, other slightly barbed; *1b* (20) longer than *1c*, *2a*, *3b*, *3c*, *4a*, *4b* (12–16) and *1a*, *3a* (4–6). Setae *3c* thickest. Pedotecta I large, concave (measured in dorsal view) and scale-like (measured in lateral view); pedotecta II smaller, trapezoid, with one pointed tip anteriorly (measured in ventral view) and scale-like (measured in lateral view). Discidia elongated, weakly triangular. Circumpedal carinae distinct.

*Anogenital region*. Two pairs of genital setae (8) setae thin, slightly barbed. One pair of aggenital setae (6–8) setae thin, smooth. One pair of anal (106–135) and two pairs of adanal setae (114–143) very long, flagellate. Often anal setae brokened, only alveoli visible. Lyrifissures *iad* not visible.

*Legs*. Median claw weakly thicker than two lateral claws; all with several minute barbs on dorsal side. Lateral claws with ventral tooth. Formulae of leg setation and solenidia: I (1–5–2–4–16) [1–2–2], II (1–5–2–4–13) [1–1–2], III (2–3–1–3–13) [1–1–0], IV (1–2–2–3–11) [0–1–0]; homology of setae and solenidia indicated in Table 1. Famulus short, straight, slightly dilated distally, truncated. Solenidia ω_2_ on tarsi I, ω_1_ and ω_2_ on tarsi II, σ on genua II, III of medium size, thickened, blunt-ended. Other solenidia long, setiform.

**Figures 45–49. F9:**
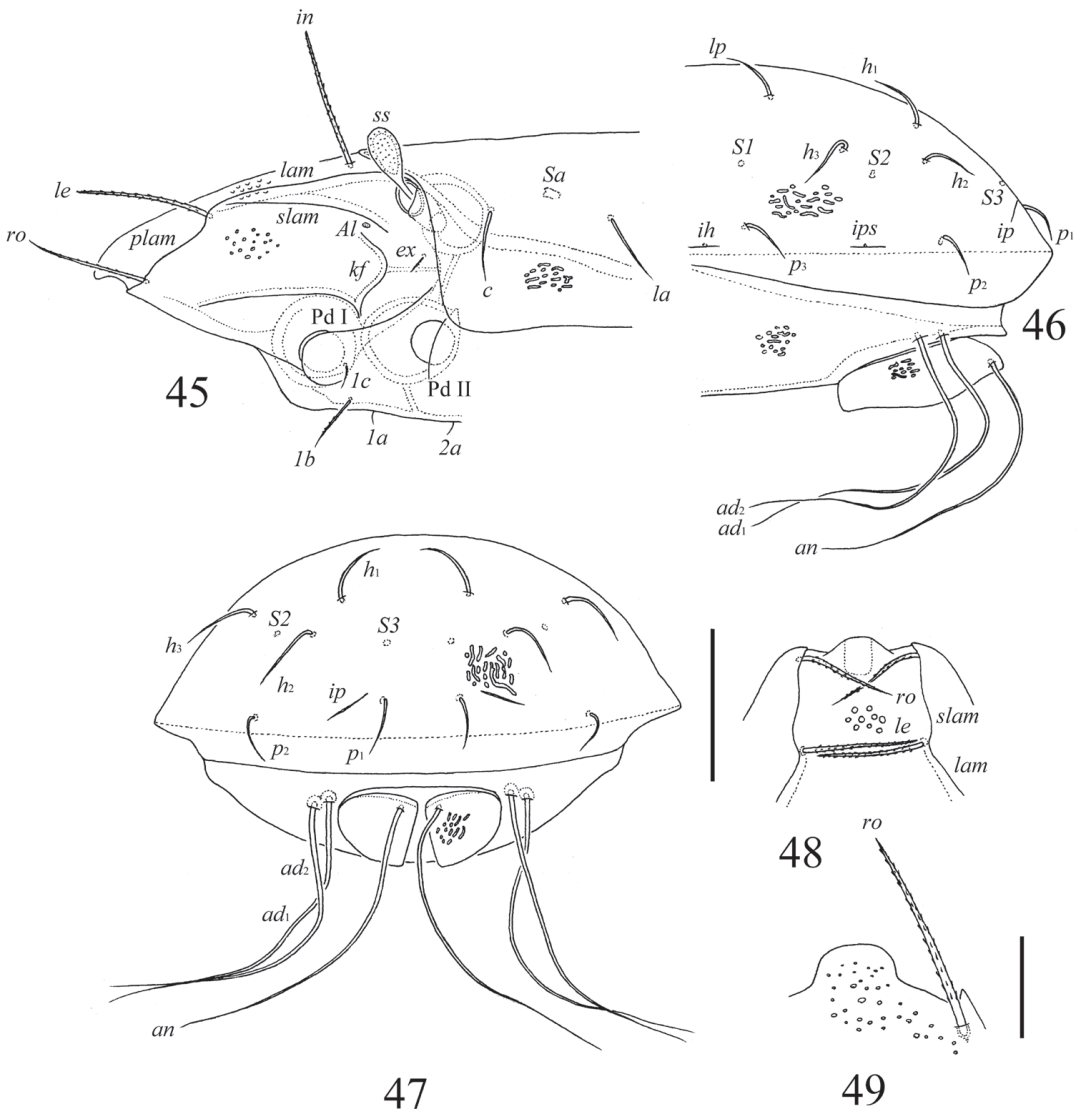
*Benoibates minimus* Mahunka, 1985, Costa Rican specimen: **45** lateral view of prodorsum and anterior part of notogaster and pteromorph (gnathosoma and legs I, II not illustrated) **46** lateral view of notogaster **47** posterior view of notogaster **48** frontal view of prodorsum **49** rostrum and right rostral seta in dissected specimen. Scale bars 50 μm (**45–48**), 20 μm (**49**).

**Figures 50–59. F10:**
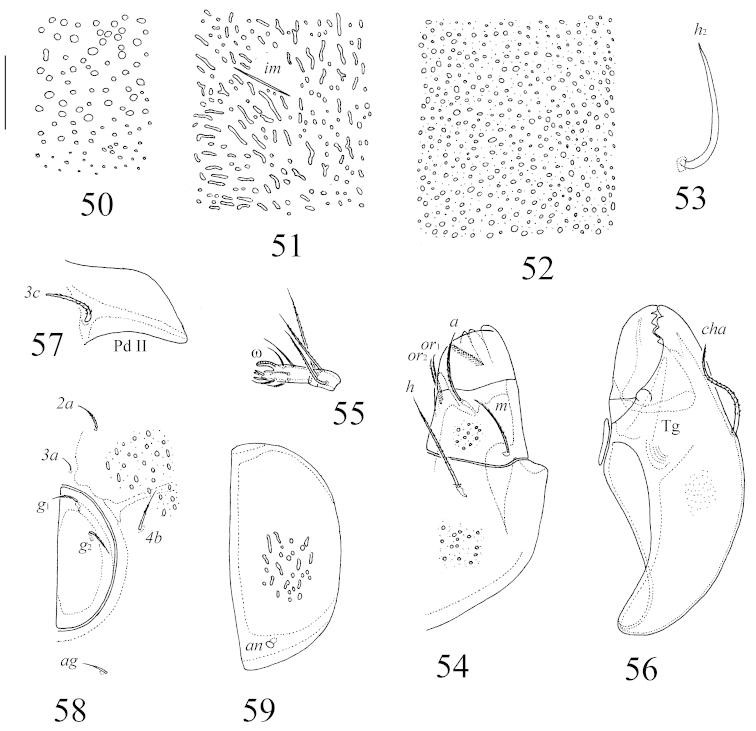
*Benoibates minimus* Mahunka, 1985, dissected Costa Rican specimen: **50** foveoles in medio-basal part of prodorsum **51** foveoles in lateral part of notogaster and lyrifissures *im* **52** foveoles in central part of anogenital region **53** notogastral seta *h*_2_ **54** left part of subcapitulum, ventral view **55** tarsus and tibia of palp **56** chelicera **57** pedotectum II and epimeral seta *3c* **58** left genital fig and epimeral setae *2a*, *3a*, *4b* **59** left anal fig. Scale bar 20 μm.

**Figures 60–61. F11:**
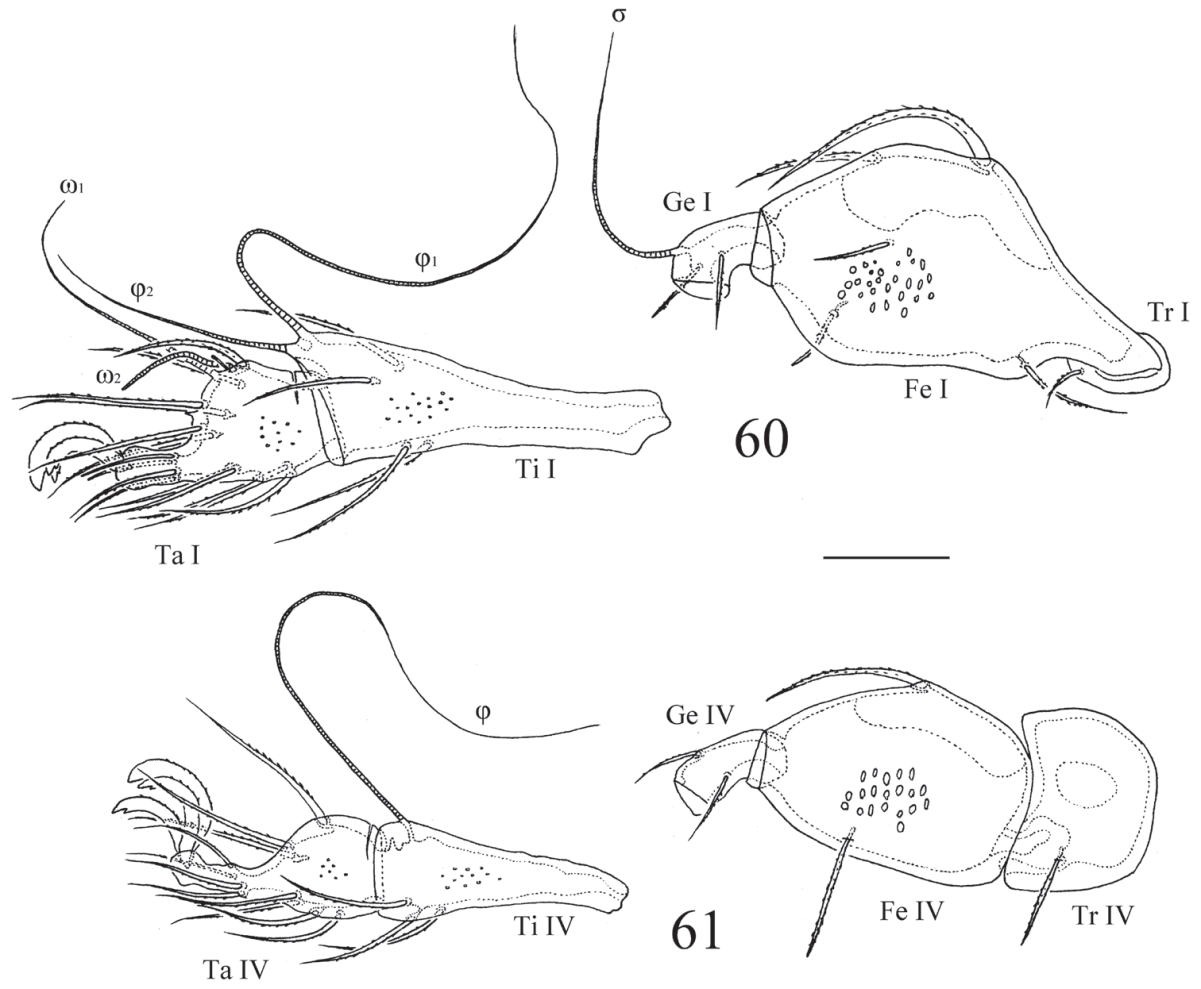
*Benoibates minimus* Mahunka, 1985, dissected Costa Rican specimen: **60** left leg I, antiaxial view **61** right leg IV, antiaxial view. Scale bar 20 μm.

**Figures 62–70. F12:**
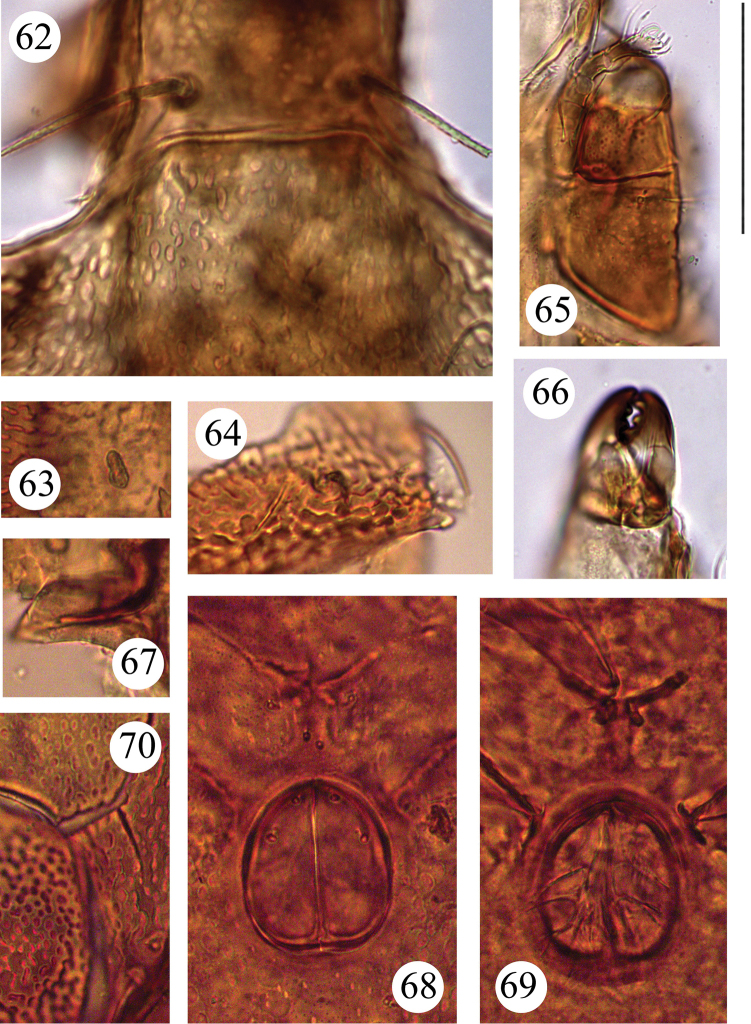
*Benoibates minimus* Mahunka, 1985, dissected Costa Rican specimen, microscope images: **62** dorsal view of basal part of prodorsum and anterior notogastral margin **63** saccule *Sa* **64** lyrifissure *ip* and foveoles in posterior part of notogaster **65** right part of subcapitulum, ventral view, and right palp **66** anterio-medial part of chelicera **67** ventral view of right pedotectum II **68** genital figs and central part of epimeral region **69** genital figs and central part of epimeral region **70** medio-lateral part of left anal fig, insertions of adanal setae, and foveoles in anogenital region. Scale bar 50 μm.

**Figures 71–74. F13:**
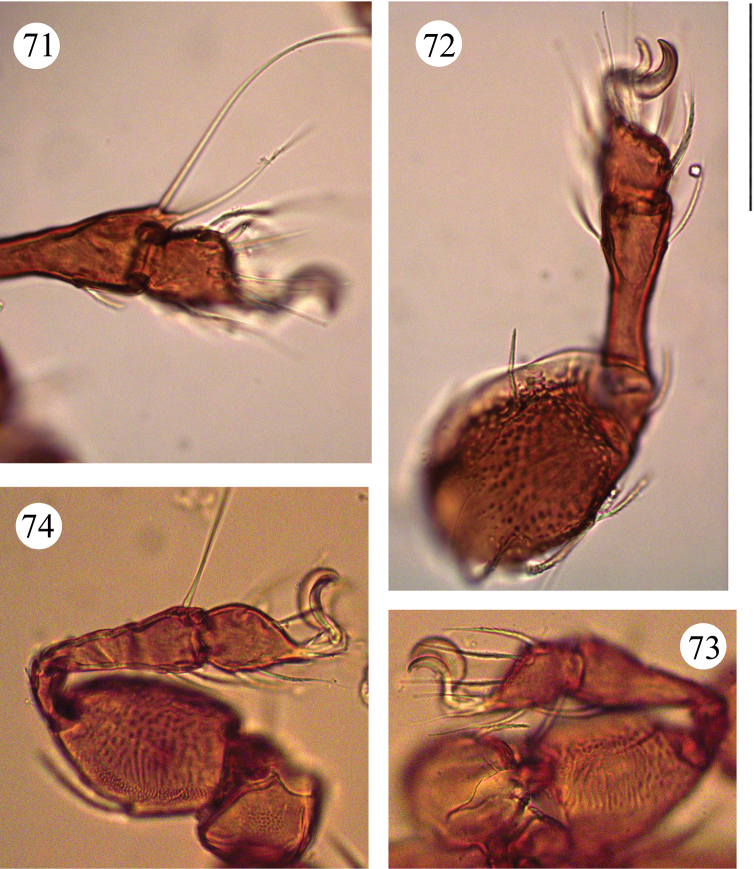
*Benoibates minimus* Mahunka, 1985, dissected Costa Rican specimen, microscope images: **71** tarsus and antero-medial part of tibia of leg I, left, paraxial view **72** leg II, left, antiaxial view **73** leg III, right, antiaxial view **74** leg IV, left, paraxial view. Scale bar 50 μm.

**Figures 75–78. F14:**
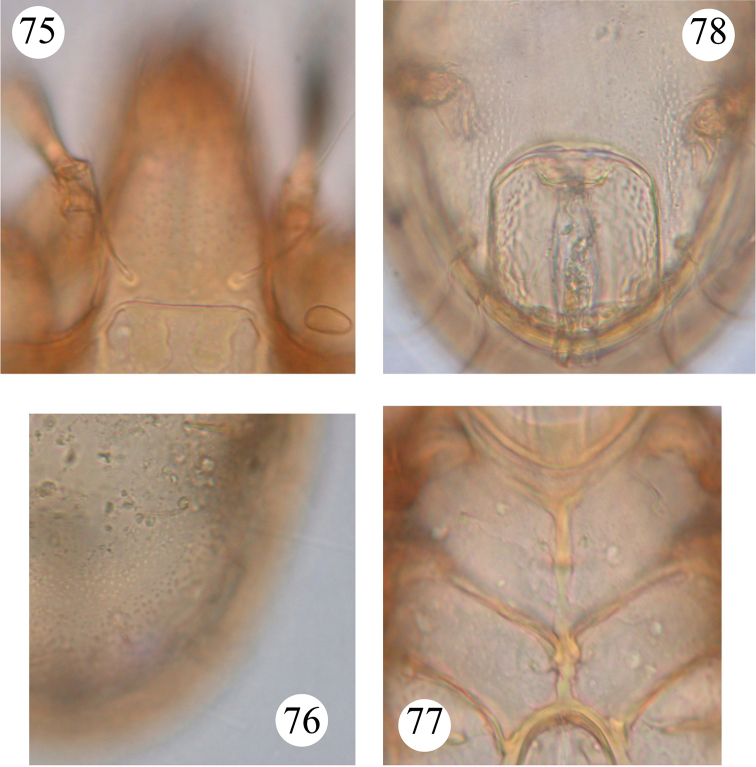
*Benoibates minimus* Mahunka, 1985, holotype, microscope images: **75** dorsal view of prodorsum and anterior part of notogaster **76** dorso-lateral part of notogaster **77** epimeral region **78** ano-adanal region.

**Figures 79–83.**
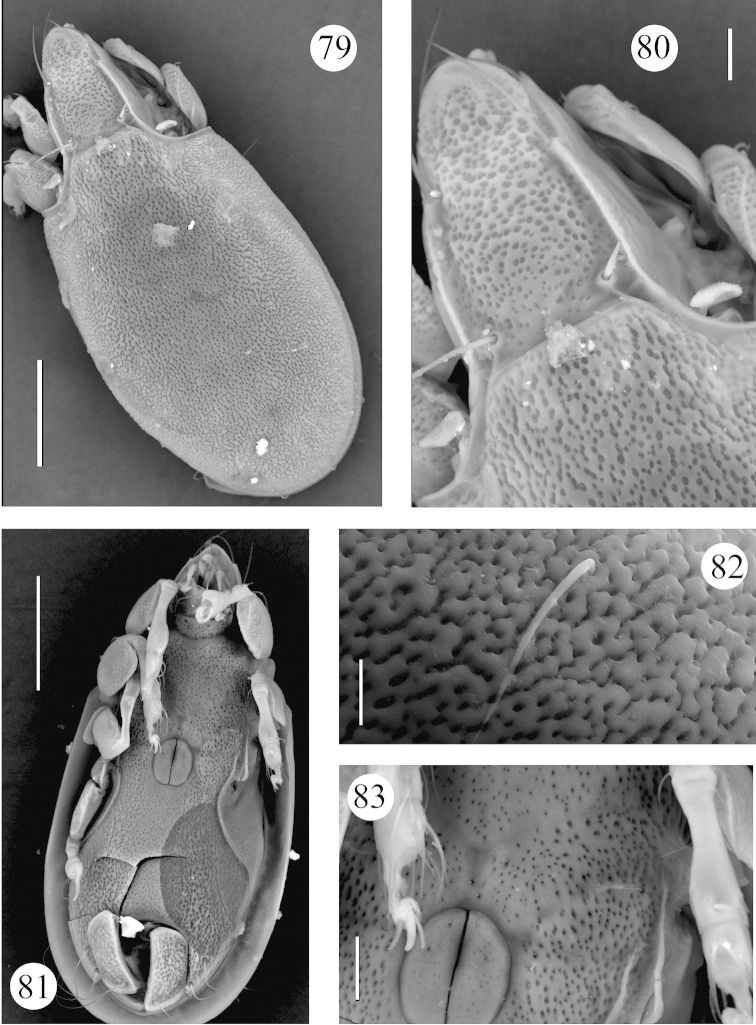
*Benoibates minimus* Mahunka, 1985, Costa Rican specimen, SEM micrographs: **79** dorsal view **80** dorsal view of prodorsum and anterior part of notogaster **81** ventral view **82** notogastral seta *h*_2_ **83** genital figs and left part of epimeral region. Scale bar 100 μm (**79, 81**), 20 μm (**80, 83**), 10 μm (**82**).

##### Remarks.

Costa Rican specimens of *Benoibates minimus* are similar in all morphological characters to Antilles specimens from the original description (Mahunka 1985).

##### Distribution.

Neotropical region.

### Key to known species of *Benoibates*

*Benoibates crinitus* (Berlese, 1910) was very poorly described by Berlese (1910), therefore we did not include this species in the key.

**Table:** 

1	Anal and adanal setae of medium size, similar to length of anal fig or shorter	2
–	Anal and adanal setae long, flagellate, longer than length of anal fig	4
2	Anal and adanal setae setiform, twice shorter than length of anal fig; body size: 374–600 × 200–330	*Benoibates juglans* (Jacot, 1938). U.S.A.
–	Anal and adanal setae flagellate, similar in length to anal fig	3
3	Interlamellar setae dilated distally; body size: 475 × 221	*Benoibates amazonicus* Balogh & Mahunka, 1969(b). Neotropical region
–	Interlamellar setae setiform; body size: 358–625 × 165–275	*Benoibates muscicola* Baranek, 1981. Argentina
4	Lamellar setae with rounded tip, interlamellar setae dilated distally; body size: 380–472 × 255–270	*Benoibates flagellifer* Balogh, 1958 (see also Balogh 1960). Angola
–	Lamellar and interlamellar setae simple, setiform	5
5	Interlamellar setae very long, reaching the rostrum; body size: 448–584 × 210–290	*Benoibates borhidii* Balogh & Mahunka, 1980. Neotropical region
–	Interlamellar setae of medium size, not reaching the rostrum	6
6	Notogaster with 11 pairs of setae (*c*_1_ present); body size: 665–680 × 339–388	*Benoibates plurisetus* Mahunka, 1984. Paraguay
–	Notogaster with 10 pairs of setae (*c*_1_ absent)	7
7	Adanal region with foveoles, forming the longitudinal lineate rows	8
–	Adanal region with foveoles, not forming the longitudinal lineate rows	9
8	Translamella present; epimeral setae *1b* considerable longer than *1c*; body size: 355–480 × 185–272	*Benoibates rugosus* Mahunka, 2001. Kenya
–	Translamella absent; epimeral setae *1b* and *1c* similar in length; body length: 375	*Benoibates marginatus* (Hammer, 1973). Polynesia
9	Apodemes 2 connected to anterior margin of genital aperture; lyrifissures *im* in transverse position; body size: 514–597 × 265–332	*Benoibates bolivianus* Balogh & Mahunka, 1969(a). Neotropical region
–	Apodemes 2 not connected to anterior margin of genital aperture; lyrifissures *im* in diagonal position	10
10	Distance *ad*_1_–*ad*_1_ equal to *ad*_2_–*ad*_2_; bothridial head small; body size: 585–647 × 369–388	*Benoibates chacoensis* Mahunka, 1984. Paraguay
–	Distance *ad*_1_–*ad*_1_ smaller than *ad*_2_–*ad*_2_; bothridial head large; body size: 344–481 × 176–249	*Benoibates minimus* Mahunka, 1985. Neotropical region

## Supplementary Material

XML Treatment for Benoibates bolivianus

XML Treatment for Benoibates minimus
